# Addressing Common Pain Syndromes in Pediatric Stem Cell Transplant: A Review

**DOI:** 10.3390/children9020139

**Published:** 2022-01-21

**Authors:** Avis Harden, Kimberly Kresta, Nelda Itzep

**Affiliations:** Pediatric Hospice and Palliative Medicine, Department of Pediatrics, University of Texas at MD Anderson Cancer Center, Houston, TX 77030, USA; KMKresta@mdanderson.org (K.K.); NPitzep@mdanderson.org (N.I.)

**Keywords:** pain, stem cell transplant, palliative, mucositis

## Abstract

Assessment and management of pain for pediatric patients receiving stem cell transplants can be challenging for a health care team. Diagnostic evaluation and interventions vary between institutions and individual provider practices. In this review, we investigate and describe approaches to pain management for the most common sources of pain in pediatric patients undergoing stem cell transplants. Mucositis pain, abdominal pain, and hemorrhagic cystitis emerged as the most frequent sources of acute pain in children during conditioning and transplantation periods. Furthermore, psychosocial distress and psychological pain or distress constitute significant components of the total pain experienced by children undergoing stem cell transplantation. We will expand upon appropriate usage and escalation of opioids, as well as complementary interventions and timely initiation of interventions, in order to help control pain in these clinical syndromes.

## 1. Introduction

Stem Cell transplantation (SCT) is a potentially life-saving procedure that can result in increased long-term survival for patients with otherwise terminal disease [1,2]. Despite its potential for decreasing mortality in life-threatening childhood disorders, SCT is an intensive and taxing procedure for patients and their families, and it is accompanied by significant pain morbidity. Patients are often subject to numerous procedures and clinical evaluations that may cause pain. SCT conditioning regimens administered prior to cell infusion often cause painful and uncomfortable sequelae for transplant recipients. Physical pain is common, but psychological pain or distress and psychosocial pain are important morbidities as well. There has been a significant amount of research and establishment of guidelines dedicated to improving the transplant process in order to maximize successful clinical outcomes and minimize morbidity and mortality. There has also been research evaluating long-term neurocognitive outcomes for pediatric patients who receive SCT [3]. This research has resulted in less toxic conditioning regimens, more uniform guidelines for patient care, and greater incorporation of supportive care measures [4,5]. Adult groups have created coordinated approaches to manage some pain syndromes in adult SCT recipients [6]. Despite advances in transplant conditioning regimens and clinical support measures and despite earlier and more frequent involvements of palliative care teams for SCT patients, few guidelines for pain management in pediatric SCT settings have been established. In the context of pediatric SCT, it is important to consider the role of a more standardized approach to palliative and supportive care measures, particularly those pertaining to management of common pediatric pain syndromes. Herein, we will review the most commonly encountered pediatric pain syndromes identified from our literature search, which includes mucositis pain, abdominal pain, bladder pain or pain related to cystitis, and psychological pain or distress.

## 2. Methods

A comprehensive search of peer-reviewed journals was completed by using key terms including “pain”, “pain management”, “stem cell transplant”, “BMT”, “pediatric bone marrow transplant pain”, “cystitis”, “mucositis”, “quality of life”, “psychosocial pain”, “psychological pain or distress”, “spiritual pain”, and “abdominal pain.” Databases searched included, but were not limited to, PubMed and Google Scholar. The reference section of each article identified was also searched in order to identify any additional articles. Both pediatric-centered and adult-centered references were considered for systematic approaches to pain management in pediatric SCT, which appear to be limited. The search process uncovered 40 articles published from 1989 to 2021.

## 3. Discussion

### 3.1. Mucositis

One of the most common side effects and sources of discomfort from SCT is mucositis. Mucositis is associated with an administration of radiotherapy and/or chemotherapy. It is the result of inflammation and ulceration of the gastrointestinal tract from damage to the epithelial mucous membranes [6]. Epithelial sloughing, inflammation, and ulceration generally occurs 4 to 5 days after administration of the conditioning regimen. It can last for at least a week to a few weeks after conditioning chemotherapy. In general, mucositis heals with immune reconstitution, as the damaged tissue recovers from the initial insult. In the time between initial insult and mucosal recovery, opioid analgesia and, in many instances, patient-controlled analgesia (PCA) are effective treatment options for SCT patients [7]. Some patients may start to experience discomfort from mucosal sloughing as early as a few days after receiving chemotherapy or radiation. As such, opioid analgesics can be initiated as soon as the patient begins make complaints related to difficulties in eating and drinking or of general oral and throat discomfort. Patients may initially only require opioid doses as needed; however, depending on the conditioning regimen, transplant type, and individual patient thresholds, as-needed opioid dosing may become insufficient for pain control. Intravenous opioids are often used as a first choice when pain is severe due to its onset of action being faster than oral opioids and due to the frequency with which SCT patients with mucositis are unable to tolerate medications by mouth (PO). Doses typically start at 0.1 mg/kg of IV morphine (or equivalent), with an initial maximum of 2 mg per dose every 2 h for an opioid-naïve individual. If a patient requires more than 6–7 as-needed doses of pain medication per 24 h, it is appropriate to consider PCA. Escalation to PCA should be determined by the provider with input from the patient or patient’s caregiver. The expediency with which a patient can receive pain management is one of several advantages of PCA, along with the potential for self-titration and the ability for patients to have control over their pain management [8]. PCA pumps provide an opioid dose on demand and, depending on the extent of pain, PCA pumps can also be programmed to provide a continuous infusion of opioids. Providers should consider each patient’s prior 24-hour opioid exposure in order to calculate the appropriate PCA dose when initiating PCA. At our institution, the initial approach to determining PCA settings and adjustments was outlined by a multidisciplinary team and was used as an institutional standard of care, as presented in Table 1. It is important to educate the caregiver, nursing staff, and patient regarding appropriate use of PCA, including opioid use only for physical pain, risks, and indicators of toxicity (e.g., somnolence, delirium, respiratory depression, and myoclonus); tempering expectations about time to and degree of pain relief; indicators of pump readiness to administer a bolus dose; and function of basal rate dosing. Generally, children younger than 6 years old may not be able to utilize PCA.; therefore, patients’ caregivers/parents or nursing staff may administer demand doses through PCA by proxy [9].

When evaluating efficacy of an active opioid regimen to determine if, when, and how PCA doses should be titrated, it is important to evaluate daily pain scores, as well as the 24-hour opioid requirement that should take into account any additional IV doses administered as needed by nurses, PCA doses received, and the basal opioid dose. The provider should also consider PCA button demands compared to delivered doses. Demand in excess of doses administered may suggest suboptimal PCA dosing, need for further education about how PCA should be used, or a need for redistribution of PCA dose and basal dose settings. Generally, PCA doses can be increased by 25% every 4 h as needed, and care should be taken to avoid rapid escalation of basal rates before a steady state is achieved (6–8 h). Discussions with patients and families should be incorporated into decision making to ensure that opioids are being used appropriately, i.e., for pain control and not for any non-pain syndrome, and also to ensure that opioids are not being used as a chemical coping mechanism to inappropriately treat illness associated psychosocial or spiritual distress.

Other evidence-based mucositis treatments to consider include good oral hygiene and mouth washes: sodium bicarbonate, magic mouthwash, and xyloxylin [10]. Magic mouthwash is an oral rinse that most commonly includes an antacid suspension and diphenhydramine, with or without added antibiotics or steroids. There does not appear to be a standardized approach for mucositis pain in pediatrics. It is imperative to work closely with patients’ transplant teams to ensure adequate and safe mucositis pain relief. Providers may also consider the suggestion of preventative measures such as palifermin or low-level light therapy [11,12,13]. A future direction of research includes manipulation of microbiome diversity in order to improve mucosal integrity [13].

### 3.2. Abdominal Pain

Acute abdominal pain is common after stem cell transplantation and may occur in varying presentations with numerous etiologies. Generalized symptoms, such as abdominal pain in any or all quadrants, fever, nausea, vomiting, anorexia, dyspepsia, diarrhea, ascites, and constipation, require more workup since they are non-specific and may not independently suggest any specific diagnosis [14,15]. These symptoms commonly represent hepatic veno-occlusive disease (VOD), graft versus host disease (GVHD), or opportunistic infection. Initial laboratory studies and clinical signs may be misleading and non-specific. It is important to conduct a proper evaluation with imaging or further testing that may include ultrasound, CT scan, gastrointestinal panel, hepatic, and pancreatic function studies. Colonoscopy during pancytopenia is usually contraindicated [14]. VOD is characterized by occlusion in small hepatic veins caused by endothelial inflammation, which results in hepatic portal hypertension. Liver ultrasound can support diagnosis by illustrating vascular changes and can assist with therapy monitoring [16,17]. Supportive care treatments include maintenance of euvolemia with diuretics; minimizing hepatotoxic medications; draining abdominal or pulmonary fluid collections that may be causing pain or dyspnea (paracentesis, and thoracentesis); and oxygen, analgesia, and defibrotide. In the setting of renal dysfunction, escalation to hemodialysis or hemofiltration may indicate maintenance of fluid balance. Intensive care might be required. Pain management with IV opioids is often needed. The degree of hepatic and/or renal insufficiency should be considered when choosing and dosing opioids. Morphine and hydromorphone should be initiated with longer dosing intervals due to slower rates of clearance in patients with hepatic insufficiency [18]. Furthermore, in patients with hepatorenal syndrome, accumulations of active glucuronidated metabolites of morphine and hydromorphone may preclude the safe use of these agents. Fentanyl, with inactive metabolites, is the preferred opioid in this setting. Another diagnosis that presents abdominal pain in transplant settings is acute intestinal GVHD. This type of GVHD typically presents in the first 100 days after stem cell transplant. CT scans may show abnormal mucosal enhancement of the entire GI tract with emphasis on the small bowel [14]. Endoscopy and biopsy of the GI tract may also be necessary and can be safely accomplished if a patient is hemodynamically stable with no evidence of hemorrhage and ANC > 500/dL and rising. Initial treatment includes systemic steroids while closely monitoring infections. Pain is generally a result of abdominal cramping and can be mitigated with hyoscyamine or dicyclomine; sometimes, warm compresses applied to the abdomen can provide comfort. Intravenous opioids may also be needed. Other causes of abdominal pain to consider include typhlitis, clostridium difficile, CMV colitis, fungal infections such as Candida albicans (most common), aspergillus, cryptococcus neoformans, and mucormycosis [14]. Investigation for fungal infection is warranted when fever persists and does not improve with antibiotic treatment. If a fungal source is identified, providers should work with their infectious disease team to determine appropriate pharmacologic intervention. As with mucositis, intravenous opioids are the mainstay of pain management for severe abdominal pain due to colitis and VOD that are provided as needed initially and, if necessary, advanced to PCA. However, as previously stated, care must be taken to consider the degree of hepatic or renal impairment and its resulting delay on clearance of opioids and their metabolites in the case of morphine and hydromorphone.

### 3.3. Hemorrhagic Cystitis

Hemorrhagic cystitis (HC), which is inflammation of the bladder mucosa resulting in bleeding within the bladder, is a complication that may be encountered in the pre-engraftment period or early post-engraftment period following bone marrow transplant. In the pre-engraftment period, HC may be secondary to the conditioning regimen, particularly when conditioning agents include total-body irradiation (TBI), cyclophosphamide, ifosfamide, and busulfan. In the early post-engraftment period, approximately 3–6 weeks after cell infusion, hemorrhagic cystitis is often caused by viral entities, specifically BK virus and, less commonly, adenovirus [19]. While there is a good amount of research regarding treatments of hemorrhagic cystitis, there is little published research describing a standardized approach for the management of pain associated with hemorrhagic cystitis [8,20,21]. Pain may be secondary to bladder inflammation, but it may also be secondary to some treatment modalities. In severe cases of hemorrhagic cystitis where continuous bladder irrigation is required, a major source of pain is the presence of an irrigation catheter. Depending on the severity of HC and the duration of symptoms, the presence of a catheter may be prolonged for several weeks. Management of this pain, which is primarily nociceptive but may also have neuropathic components, can be challenging. Resolution of contributing factors can result in the amelioration of bladder pain, but a viscera-directed approach to bladder inflammatory pain is opioid therapy, which can be titrated to patient need, and may require PCA use. At our institution, the initial approach to determining PCA settings and adjustments is outlined in Table 1. It is important to note that a patient-controlled dose, demand, or “button” dose may most expeditiously eliminate acute pain flares occurring when a nurse may not be readily available to administer an as-needed (PRN) dose [22]. In conjunction with a direct approach to the pain itself, one should consider addressing mechanical factors that may exacerbate pain. These include bladder spasms for which hyoscyamine or dicyclomine can be effective. Rectally administered Belladonna and Opium (B&O) suppositories can provide additional relief for bladder spasms and are used routinely after bladder surgery in children, and they have been shown to be efficacious in routine managements of bladder pain [23]. In the SCT setting, however, white blood cell and platelet counts must be adequate for medication administration per rectum. Oxybutynin chloride may also be considered to reduce urinary frequency, and short-course phenazopyridine may be considered for its local analgesic effects. However, phenazopyridine will obscure response to therapy given its discoloration of urine and can itself result in kidney toxicity. While excessive hydration should be limited, particularly in transplant contexts when fluid overload can increase morbidity, generally, fluid intake should be adequate for renal perfusion [24]. Outside of pharmacologic intervention, patients should be encouraged to use distraction techniques to combat pain and distress caused by bladder pain and catheter placement.

### 3.4. Psychological Pain or Distress

Bone marrow transplantation can be exceptionally challenging for pediatric patients physically and also psychologically. The traditional perception of pain characterizes the phenomenon to be primarily physical. In reality, patients experience pain not only as physical manifestations but also as psychological manifestations [25]. Psychological pain or distress, while described as far back as the 1970s when SCT was nascent, has only over the last 10–20 years begun to be a focus of research study questions [26]. Psychological distress may be present in pediatric patients from the time of the first family discussion about SCT as a clinical intervention [26]. These children, who often have previously endured numerous medical interventions, are often burdened with financial, psychosocial, emotional, and mental stressors related to their illness prior to considerations of SCT. As such, SCT places the patient and their family at risk for psychological maladjustment and dramatic decrease in quality of life [27]. Psychological distress continues throughout transplantation; in particular, pediatric patients have significant psychosocial difficulties in the first year following SCT [28,29]. Symptoms of psychological distress, or psychological pain or distress, during and after SCT, can range from general stress to depression, anxiety, and behavioral issues [25,26]. Behavioral issues may manifest as a loss of developmental milestones in younger patient populations or as outbursts of anger and demand for control in older patient populations [26]. If left unaddressed, psychological pain or distress can result in post-traumatic stress disorder (PTSD) and lower health-related quality of life (HRQoL) throughout and acutely following bone marrow transplantation [30,31]. Family cohesiveness is a psychosocial predictor of distress in pediatric patients undergoing stem cell transplantation [27]. Healthy family functioning and higher levels of family cohesiveness may serve as protective factors for a child’s psychological adjustment during and following SCT [27]. Understanding this predictor early in the transplant course can guide expectations for quality-of-life outcomes. Early intervention by pediatric palliative care services can help with patient and family coping and is beneficial in maximizing end-of-life planning [5,32]. Early intervention can also help achieve patient and family quality-of-life goals and address worries in order to aid psychological stress management throughout the transplant process [32]. We, as have others in the field, propose early consultation of pediatric palliative care teams as a standard of care in the setting of bone marrow transplantation [33]. Interdisciplinary communication is the foundation for ensuring prompt assessment and management of various manifestations of psychosocial distress. Similar to the case at many institutions, at our institution, all patients and families receive social work referral for necessary social support services and counseling [34]. Mood disorders, anxiety, and sleep disorders, all of which are often present in pediatric SCT patients, are typically managed by palliative care services, with more challenging cases referred to psychiatry. Patients are also routinely referred to psychologists for assistance with coping, as well as for supportive, cognitive, and behavioral therapy for changes in mood and anxiety [35]. Anxiety is often managed acutely with common anxiolytics including lorazepam and, in a more long-term approach, with SSRIs. Depression is often managed pharmacologically with an SSRI, provided that there are no clinical contraindications, and the family is amenable. Providers may consider implementation of additional non-pharmacologic interventions including music therapy, distraction techniques, guided imagery, and relaxation training [36]. Although it is not commonly addressed in the literature we reviewed, spiritual distress and how it contributes to psychological pain or distress should be considered for pediatric patients receiving SCT [37,38,39]. Screening tools for pediatric patients can be used to assess spiritual distress [39]. Incorporation of the previously listed interdisciplinary non-pharmacologic therapies; provision of community spiritual resources; meditation; and enlistment of chaplaincy at the patient and family’s discretion can all aid in treating spiritual distress [38].

It is important to acknowledge that there is a relationship between psychological distress and physical pain, in which one can magnify the other. Therefore, effective management of both entities is optimum for improving the overall pain experience of pediatric SCT patients [40].

## 4. Conclusions

SCT is a complex and trying procedure that, while being potentially lifesaving, can cause pediatric patients an immense amount of pain in various domains. Pain syndromes such as mucositis, abdominal pain, and hemorrhagic cystitis, as well as psychosocial distress, are the most common sources of pain experienced during SCT, and syndromes should be addressed thoroughly when caring for these children. Although mucositis and abdominal pain management have been well researched, hemorrhagic cystitis pain has been less well studied. In addition, while intravenous opioids remain the backbone to the management of pain during transplantation, a systematic, multifaceted approach to managing these syndromes can deliver improved results. Addressing physical pain and psychosocial distress will provide the best outcomes, as each can attenuate and magnify the other. Ultimately, this comprehensive approach to total pain can result in a more positive experience for patients, as well as their families, as they undergo SCT.

## Figures and Tables

**Table 1:** Pediatric Patient Controlled Analgesia (PCA) Tip Sheet.

Opioid	Demand (PCA) Dose (Dose Range)	Lock-Out Interval (Minutes)	1-h Dose Limit (Optional)	Continuous Rate (Basal)
Morphine (mg)	0.01–0.03 mg/kg	10–30 min	5 doses per hour	0–0.03 mg/kg/h
Hydromorphone (mg)	0.003–0.004 mg/kg	10–30 min	5 doses per hour	0–0.004 mg/kg/h
Fentanyl (mcg)	0.5–1 mg/kg	10–30 min	5 doses per hour	0–0.5 mcg/kg/h

**1. Opioid Naïve Patients**: (a). Patient should be alert and able to demonstrate ability to administer demand dose for pain. If there are concerns about altered mental status or significant anxiety, consider specialty consultation with psychology, psychiatry, interventional pain service, pediatric palliative medicine, anesthesiology, or pediatric intensive care as needed and/or available in your institution. (b). Carefully consider adding continuous (basal) rate after 12–24 h if using frequent demand doses or if pain is uncontrolled. Suggested basal dose is 30–50% of average hourly dose. * Example: The 12-h total morphine demand dose is 20 mg, calculate continuous dose as 20 mg/12 h = 1.7 mg/h then 1.7 × 0.3 (30%) = 0.5 mg/h basal rate. (c). Depending on pain severity, a patient may also require a nurse bolus dose, which is a bolus dose (larger than the demand dose) that is available every 1–3 h (interval as per physician discretion taking into account pain etiology, dose, and pain severity). It is administered by a nurse after nursing assessment of pain level, vital signs, mental status, and physical exam. **2. Opioid tolerant patients (currently receiving opioid therapy)** PCA orders should take into account the patient’s current opioid regimen, clinical situation (severity and etiology of the pain, side effects from opioids, baseline drowsiness, need for opioid rotation). If there are significant side effects, including drowsiness, confusion, respiratory or central nervous system concerns, we recommend consultation with specialty services as listed above in 1. (a). (a). Calculate total dose of opioid (scheduled and breakthrough doses) used in the previous 24-hour period. (b). Convert to morphine equivalent daily dose. (c). Use your institutional equianalgesic opioid dose conversion table to calculate dose of IV opioid being considered for PCA. Decrease dose by 30–50% to account for lack of complete cross tolerance to obtain new IV dose. d. Divide total by 2 and put ½ into the continuous rate and ½ in the PCA button. e. If patient requires a “rescue” opioid dose, the RN may give 2× the demand button dose. * Example: If daily total for morphine IV is 20 mg, continuous rate would be 0.4 mg/h (10 mg/24 h), demand dose would be 0.4mg Q10–30 min, and nurse bolus dose would be 0.8 mg (2× demand dose) Q1–3 h.

## References

[1] Keating A.K., Langenhorst J., Wagner J.E., Page K.M., Veys P., Wynn R.F., Stefanski H., Elfeky R., Giller R., Mitchell R. (2019). The influence of stem cell source on transplant outcomes for pediatric patients with acute myeloid leukemia. Blood Adv..

[2] Bhojwani D., Pui C.H. (2013). Relapsed childhood acute lymphoblastic leukaemia. Lancet Oncol..

[3] Barrera M., Atenafu E. (2008). Cognitive, educational, psychosocial adjustment and quality of life of children who survive hematopoietic SCT and their siblings. Bone Marrow Transplant..

[4] Lafond D.A., Patterson Kelly K., Hinds P.S., Sill A., Michael M. (2015). Establishing Feasibility of Early Palliative Care Consultation in Pediatric Hematopoietic Stem Cell Transplantation. J. Pediatr. Oncol. Nurs..

[5] Mekelenkamp H., Schröder T., Trigoso E., Hutt D., Galimard J.E., Kozijn A., Dalissier A., Gjergji M., Liptrott S., Kenyon M. (2021). Specialized Pediatric Palliative Care Services in Pediatric Hematopoietic Stem Cell Transplant Centers. Children.

[6] Ma J.D., El-Jawahri A.R., LeBlanc T.W., Roeland E.J. (2018). Pain Syndromes and Management in Adult Hematopoietic Stem Cell Transplantation. Hematol. Oncol. Clin. N. Am..

[7] Vasquenza K., Ruble K., Chen A., Billett C., Kozlowski L., Atwater S., Kost-Byerly S. (2015). Pain Management for Children during Bone Marrow and Stem Cell Transplantation. Pain Manag. Nurs..

[8] Liem X., Saad F., Delouya G. (2015). A Practical Approach to the Management of Radiation-Induced Hemorrhagic Cystitis. Drugs.

[9] Anghelescu D.L., Burgoyne L.L., Oakes L.L., Wallace D.A. (2005). The safety of patient-controlled analgesia by proxy in pediatric oncology patients. Anesth. Analg..

[10] Brown T.J., Gupta A. (2020). Management of Cancer Therapy-Associated Oral Mucositis. JCO Oncol. Pract..

[11] Sung L., Robinson P., Treister N., Baggott T., Gibson P., Tissing W., Wiernikowski J., Brinklow J., Dupuis L.L. (2017). Guideline for the prevention of oral and oropharyngeal mucositis in children receiving treatment for cancer or undergoing haematopoietic stem cell transplantation. BMJ Support. Palliat. Care.

[12] Daugelaite G., Užkuraitytė K., Jagelavičienė E., Filipauskas A. (2019). Prevention and Treatment of Chemotherapy and Radiotherapy Induced Oral Mucositis. Medicina.

[13] Bowen J.M., Wardill H.R. (2017). Advances in the understanding and management of mucositis during stem cell transplantation. Curr Opin. Support. Palliat. Care.

[14] Hordonneau C., Montoriol P.F., Guièze R., Garcier J.M., Da Ines D. (2013). Abdominal complications following neutropenia and haematopoietic stem cell transplantation: CT findings. Clin. Radiol..

[15] Barker C.C., Anderson R.A., Sauve R.S., Butzner J.D. (2005). GI complications in pediatric patients post-BMT. Bone Marrow Transplant..

[16] Chan S.S., Colecchia A., Duarte R.F., Bonifazi F., Ravaioli F., Bourhis J.H. (2020). Imaging in Hepatic Veno-Occlusive Disease/Sinusoidal Obstruction Syndrome. Biol. Blood Marrow Transplant..

[17] Bonifazi F., Barbato F., Ravaioli F., Sessa M., Defrancesco I., Arpinati M., Cavo M., Colecchia A. (2020). Diagnosis and Treatment of VOD/SOS After Allogeneic Hematopoietic Stem Cell Transplantation. Front. Immunol..

[18] Bosilkovska M., Walder B., Besson M., Daali Y., Desmeules J. (2012). Analgesics in patients with hepatic impairment: Pharmacology and clinical implications. Drugs.

[19] Zaia J., Baden L., Boeckh M.J., Chakrabarti S., Einsele H., Ljungman P.P., McDonald G.B., Hirsch H. (2009). Viral disease prevention after hematopoietic cell transplantation. Bone Marrow Transplant..

[20] Corman J.M., McClure D., Pritchett R., Kozlowski P., Neil B., Hampson N.B. (2003). Treatment of radiation induced hemorrhagic cystitis with hyperbaric oxygen. J. Urol..

[21] Hassan Z. (2011). Management of refractory hemorrhagic cystitis following hematopoietic stem cell transplantation in children. Pediatr. Transplant..

[22] Anghelescu D.L., Snaman J.M., Trujillo L., Sykes A.D., Yuan Y., Baker J.N. (2015). Patient-controlled analgesia at the end of life at a pediatric oncology institution. Pediatr. Blood Cancer.

[23] Duckett J.W., Cangiano T., Cubina M., Howe C., Cohen D. (1997). Intravesical morphine analgesia after bladder surgery. J. Urol..

[24] Bosch P.C., Bosch D.C. (2014). Treating interstitial cystitis/bladder pain syndrome as a chronic disease. Rev. Urol..

[25] Packman W., Weber S., Wallace J., Bugescu N. (2010). Psychological effects of hematopoietic SCT on pediatric patients, siblings and parents: A review. Bone Marrow Transplant..

[26] Pot-Mees C. (1989). The Psychosocial Effects of Bone Marrow Transplantation in Children.

[27] Barrera M., Boyd-Pringle L.-A., Sumbler K., Saunders F. (2000). Quality of life and behavioral adjustment after pediatric bone marrow transplantation. Bone Marrow Transplant..

[28] Phipps S., Brenner M., Heslop H., Krance R., Jayawardene D., Mulhern R. (1995). Psychological effects of bone marrow transplantation on children and adolescents: Preliminary report of a longitudinal study. Bone Marrow Transplant..

[29] Chang G., Ratichek S.J., Recklitis C., Syrjala K., Patel S.K., Harris L., Rodday A.M., Tighiouart H., Parsons S.K. (2012). Children’s psychological distress during pediatric HSCT: Parent and child perspectives. Pediatr. Blood Cancer.

[30] Felder-Puig R., di Gallo A., Waldenmair M., Norden P., Winter A., Gadner H., Topf R. (2006). Health-related quality of life of pediatric patients receiving allogeneic stem cell or bone marrow transplantation: Results of a longitudinal, multi-center study. Bone Marrow Transplant..

[31] Clarke S.A., Eiser C., Skinner R. (2008). Health-related quality of life in survivors of BMT for paediatric malignancy: A systematic review of the literature. Bone Marrow Transplant..

[32] Levine D.R., Van Noy K., Talleur A.C., Snyder A., Kaye E.C., Baker J.N. (2020). Thoughts from the threshold: Patient and family hopes, fears, values, and goals at the onset of pediatric hematopoietic cell transplantation. Bone Marrow Transplant..

[33] Levine D.R., Baker J.N., Wolfe J., Lehmann L.E., Ullrich C. (2017). Strange Bedfellows No More: How Integrated Stem-Cell Transplantation and Palliative Care Programs Can Together Improve End-of-Life Care. J. Oncol. Pract..

[34] Wiener L., Kazak A.E., Noll R.B., Patenaude A.F., Kupst M.J. (2015). Standards for the Psychosocial Care of Children with Cancer and Their Families: An Introduction to the Special Issue. Pediatr Blood Cancer.

[35] Barker M.M., Beresford B., Bland M., Fraser L.K. (2019). Prevalence and Incidence of Anxiety and Depression Among Children, Adolescents, and Young Adults with Life-Limiting Conditions: A Systematic Review and Meta-analysis. JAMA Pediatr..

[36] Fisher E., Law E., Dudeney J., Palermo T.M., Stewart G., Eccleston C. (2018). Psychological therapies for the management of chronic and recurrent pain in children and adolescents. Cochrane Database Syst. Rev..

[37] Puchalski C., Ferrell B., Virani R., Otis-Green S., Baird P., Bull J., Chochinov H., Handzo G., Nelson-Becker H., Prince-Paul M. (2009). Improving the quality of spiritual care as a dimension of palliative care: The report of the Consensus Conference. J. Palliat. Med..

[38] Anandarajah G., Hight E. (2001). Spirituality and medical practice: Using the HOPE questions as a practical tool for spiritual assessment. Am. Fam. Physician.

[39] Snyder C.R., Hoza B., Pelham W.E., Rapoff M., Ware L., Danovsky M., Highberger L., Rubinstein H., Stahl K.J. (1997). The development and validation of the Children’s Hope Scale. J. Pediatr. Psychol..

[40] Plummer K., McCarthy M., McKenzie I., Newall F., Manias E. (2021). Experiences of Pain in Hospitalized Children During Hematopoietic Stem Cell Transplantation Therapy. Qual. Health Res..

